# 3D Printing of Biopolymer-Based Scaffolds for Bone Tissue Engineering: Materials, Fabrication, and Translational Strategies

**DOI:** 10.3390/molecules31132206

**Published:** 2026-06-23

**Authors:** Yeajin Song, Hongyoon Kim, Seunghun S. Lee

**Affiliations:** Department of Biomedical Engineering, Dongguk University, Seoul 04620, Republic of Korea; songstar2024@gmail.com (Y.S.); khy9842@dgu.ac.kr (H.K.)

**Keywords:** biopolymer, 3D printing, bioprinting, bone tissue engineering, scaffold, hydrogel, bioceramic composite, regenerative medicine

## Abstract

Bone defects from trauma, tumour resection, infection, and degenerative disease remain a major clinical burden, and autografts face limitations of supply and donor-site morbidity. Three-dimensional (3D) printing offers a route to patient-specific, architecturally defined bone scaffolds, while biopolymers from natural sources provide biodegradability, biocompatibility, and extracellular matrix-mimicking cues consistent with sustainable, green biomaterials science. This review synthesises recent progress in 3D printing of biopolymer-based scaffolds for bone tissue engineering. We first examine the principal feedstocks—alginate, gelatin and gelatin methacryloyl, collagen, chitosan, silk fibroin, cellulose, and microbial polyesters—and their preparation, crosslinking chemistry, and printability. We then compare extrusion, light-based, and indirect printing technologies and the process–property relationships governing resolution, mechanical competence, and cell viability. Composite and functionalisation strategies, including biopolymer–bioceramic hybrids and controlled delivery of growth factors and antimicrobial agents, are analysed as routes to osteoinduction, vascularisation, and infection control. Finally, we evaluate translational performance in preclinical models and outline central challenges of vascularisation, mechanical–degradation matching, scalability, and regulatory standardisation. Biopolymer 3D printing is positioned as a ve rsatile, sustainable platform whose clinical maturation depends on integrated material, structural, and biological design.

## 1. Introduction

Bone is a dynamic, hierarchically organised composite of mineralised collagen fibrils, non-collagenous proteins, and a richly vascularised cellular network of osteoblasts, osteocytes, and osteoclasts that continuously remodels in response to mechanical and metabolic demand. This architecture endows bone with a notable intrinsic capacity for self-repair, such that most fractures heal without scar formation. Nevertheless, defects that exceed a critical size—produced by high-energy trauma, tumour resection, osteomyelitis, congenital malformation, or revision surgery—surpass this regenerative threshold and cannot heal spontaneously, instead progressing to non-union or fibrous infill unless reconstructed [1]. The clinical and economic scale of this problem is substantial: bone is among the most frequently transplanted tissues, and the ageing of the global population is increasing the incidence of osteoporotic, peri-prosthetic, and revision-related defects [2].

Autologous bone grafting remains the clinical reference standard because it supplies osteogenic cells, osteoinductive factors, and an osteoconductive mineral matrix simultaneously, together with immunological compatibility. Its use is nonetheless constrained by finite supply, donor-site pain and morbidity, prolonged operative time, and unpredictable graft resorption [1,2]. Allografts mitigate the supply problem but introduce risks of immunogenicity, disease transmission, and inconsistent remodelling, while permanent metallic implants—although mechanically robust—do not biologically integrate, can shield the surrounding bone from physiological loading and thereby promote peri-implant resorption, and may eventually require revision [1]. These collective shortcomings have motivated a sustained search for engineered graft substitutes that recapitulate the structure and biology of native bone while remaining reproducibly manufacturable, sustainable, and adaptable to individual patient anatomy [3].

Tissue engineering addresses this need through the rational combination of scaffolds, cells, and bioactive signals to guide functional tissue formation. Within this triad, the scaffold occupies a central role as a temporary, instructive template for regeneration. An effective bone scaffold must satisfy a demanding and partly conflicting set of criteria: an interconnected, porous architecture that permits cell infiltration, nutrient and waste exchange, and vascular ingrowth; transient mechanical support matched to the loads of the host site; a surface chemistry and topography that promote cell attachment, proliferation, and osteogenic differentiation; and a degradation profile synchronised with new-tissue deposition so that load is gradually transferred to regenerating bone [2,4]. Achieving these properties simultaneously is non-trivial, and the relative weighting of each requirement varies with anatomical site, defect geometry, and patient factors.

Conventional scaffold fabrication—solvent casting and particulate leaching, gas foaming, freeze-drying, electrospinning, and thermally induced phase separation—can generate porous constructs but affords only stochastic control over pore size, shape, interconnectivity, and spatial distribution, and provides essentially no control over patient-specific external geometry [4]. As a result, such scaffolds are poorly suited to the complex, irregular defects encountered in craniomaxillofacial and load-bearing reconstruction, where precise anatomical fit is essential for both mechanical function and aesthetic outcome.

Three-dimensional (3D) printing, or additive manufacturing, overcomes these constraints by building constructs layer-by-layer from a digital model, which can be derived directly from a patient’s computed-tomography or magnetic-resonance data to yield an exact anatomical replica [3,5]. The technology grants deterministic, simultaneous control over macroscopic shape, internal pore network, porosity gradients, strut geometry, and the spatial placement of multiple materials and cell populations within a single construct. It has been applied across the full spectrum of bone-relevant biomaterials, from thermoplastics and bioceramics to soft hydrogels, and a wide range of complementary modalities—extrusion, vat photopolymerisation, powder-bed fusion, and material jetting—now coexist within the bone-engineering toolbox [4,6]. When the printed feedstock is a cell-laden hydrogel, the process is termed bioprinting, enabling living cells to be positioned within an instructive matrix during fabrication rather than seeded passively afterward, and thereby supporting the construction of heterogeneous, biomimetic tissue analogues [5,7].

The choice of feedstock is decisive for both manufacturability and biological outcome. Biopolymers—macromolecules sourced from animals, plants, algae, and microorganisms—are particularly attractive for bone scaffolds because they are inherently biodegradable and biocompatible; frequently display intrinsic cell-recognition motifs; and align with the principles of green, sustainable materials chemistry that increasingly guide biomaterials’ development and that frame the present discussion within the broader field of biopolymer-based materials [8,9]. Structural proteins such as collagen, gelatin, and silk fibroin, and polysaccharides such as alginate, chitosan, and cellulose can be processed under mild, aqueous, and often cytocompatible conditions, and their degradation products are typically resorbed or metabolised through physiological pathways rather than accumulating as persistent foreign material [10,11]. Their principal limitations—modest and batch-variable mechanical strength, composition dependent on natural source, and degradation kinetics difficult to tune independently of stiffness—are routinely mitigated by chemical modification, blending, and reinforcement with inorganic phases, producing composite inks that marry the bioactivity of the organic phase with the stiffness and osteoconductivity of the mineral phase, much as native bone itself is organised [12,13].

**Figure 1:**
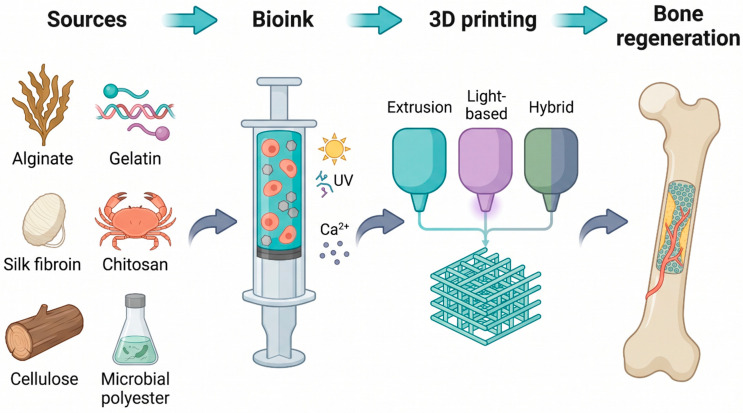
Conceptual overview: From sustainable biopolymer sources and ink formulation, through 3D printing modalities, to architecturally defined scaffolds and bone regeneration.

It is worth situating this design chain within the biology it serves. Physiological bone repair proceeds through overlapping phases—haematoma and inflammation, soft and hard callus formation, and protracted remodelling by coordinated osteoclast–osteoblast basic multicellular units—each governed by a defined sequence of signalling molecules and by mechanical cues. A successful scaffold does not merely fill a void; it must participate in this orchestrated programme, presenting the right cues at the right time and ceding space and load to regenerating tissue as it matures [2,4]. The recurring difficulty in the field is that the properties favouring one phase often oppose another: rapid degradation aids remodelling but undermines early mechanical support, while dense mineral reinforcement improves stiffness but can impede cell infiltration and vascular ingrowth. Three-dimensional printing is valuable precisely because it allows these competing requirements to be resolved spatially and temporally within a single, anatomically exact construct, and biopolymers are valuable because they offer a sustainable, biologically literate substrate on which to build (Figure 1) [3,8].

A further motivation for focusing on biopolymers specifically, rather than on 3D printing of bone scaffolds in general, is conceptual coherence. Synthetic thermoplastics such as polycaprolactone and poly(lactic-co-glycolic acid) and inert metals dominate parts of the field, but they engage the host largely as passive structural fillers, whereas biopolymers actively participate in the biology of repair through cell-recognition motifs, enzyme-cleavable backbones, and physiologically resorbable degradation products [8,10]. This biological literacy is precisely what makes natural polymers attractive as bioinks, in which cells are embedded during fabrication and must find an immediately hospitable matrix [5,7]. At the same time, the field has matured from a focus on demonstrating printability toward a more demanding standard in which scaffolds must be reproducible, sterilisable, mechanically credible, and supported by in vivo evidence, a shift that frames much of the analysis in this review [4,14]. We therefore treat biopolymer chemistry, printing process, and biological function not as separate topics but as a single coupled design problem, and we return repeatedly to the trade-offs that arise when an improvement along one axis degrades another.

This review surveys the State of the Art in 3D printing of biopolymer-based scaffolds for bone tissue engineering, organised around the design chain that links material to clinical function. Section 2 examines the major biopolymer feedstocks, their preparation, and their printability. Section 3 compares the principal printing technologies and the process–property relationships that determine scaffold quality. Section 4 analyses composite and functionalisation strategies for osteoinduction, vascularisation, and infection control. Section 5 evaluates applications and preclinical translation across defect types, and Section 6 discusses outstanding challenges and future directions. Throughout, we emphasise a recurring theme: that material chemistry, fabrication process, and biological design are interdependent and must be co-developed, rather than optimised in isolation, to advance clinically meaningful bone regeneration.

## 2. Biopolymer Feedstocks: Preparation, Properties, and Printability

The performance of a printed bone scaffold begins with the feedstock. An ideal biopolymer ink must satisfy two partly conflicting demands: it must flow or cure reliably during printing while retaining shape fidelity afterward, and it must thereafter present a biologically permissive, ideally osteoinductive, microenvironment [9,15]. These requirements are governed by molecular weight, concentration, charge, and crosslinking chemistry, and the major biopolymer classes balance them differently. Understanding their preparation and gelation mechanisms is therefore prerequisite to rational ink design and explains why most successful bone inks are blends rather than single components. The principal feedstocks and their characteristics are summarised in Table 1.

**Figure 2:**
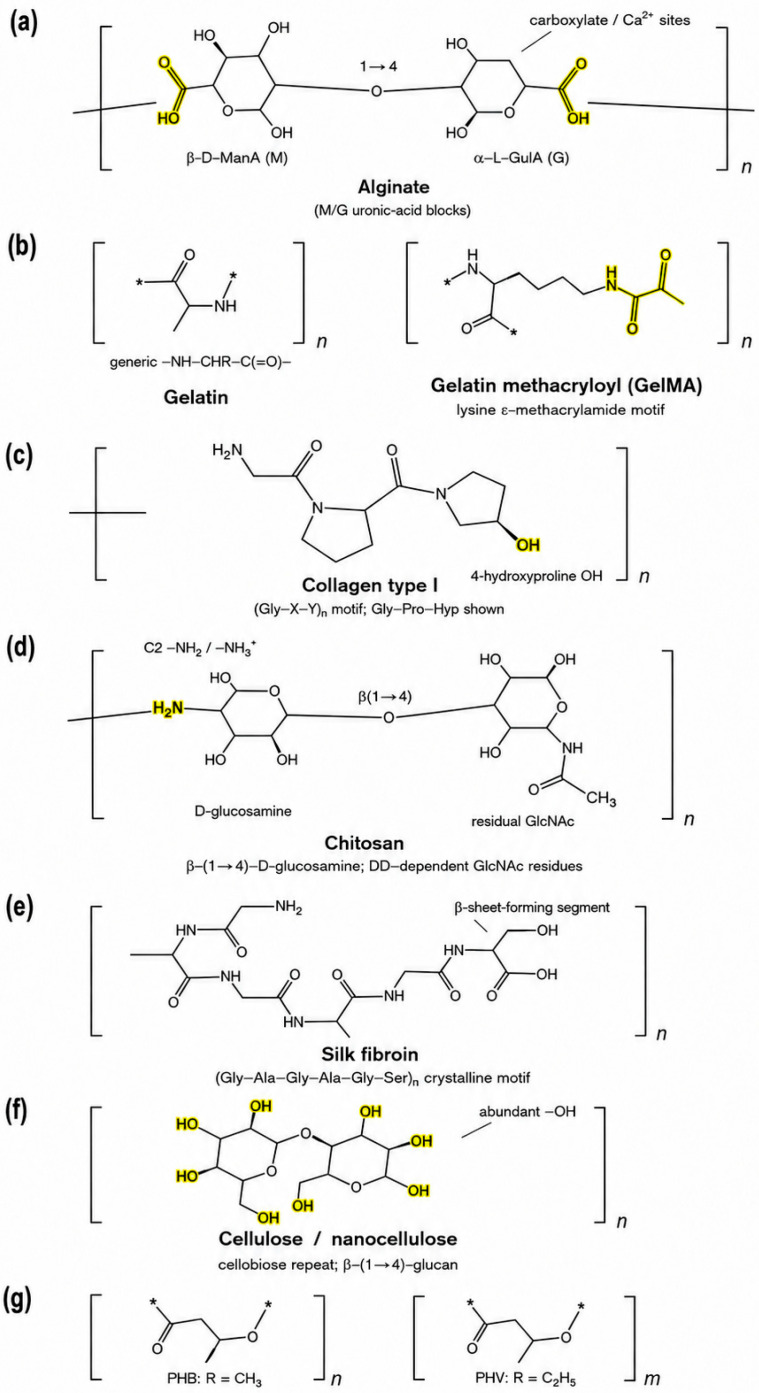
Chemical structures of the principal natural biopolymers used in 3D-printed bone scaffolds: (**a**) alginate, with β-D-mannuronate (M) and α-L-guluronate (G) blocks and Ca^2+^ “egg-box” crosslinking; (**b**) gelatin and gelatin methacryloyl (GelMA); (**c**) collagen type I; (**d**) chitosan; (**e**) silk fibroin ([Gly-Ala-Gly-Ala-Gly-Ser]_n_ crystalline motif); (**f**) cellulose/nanocellulose (β-(1→4)-glucan); and (**g**) microbial polyhydroxyalkanoates (PHB, R = CH_3_; PHV, R = C_2_H_5_). Functional groups governing crosslinking, solubility, and printability are highlighted.

**Figure 3:**
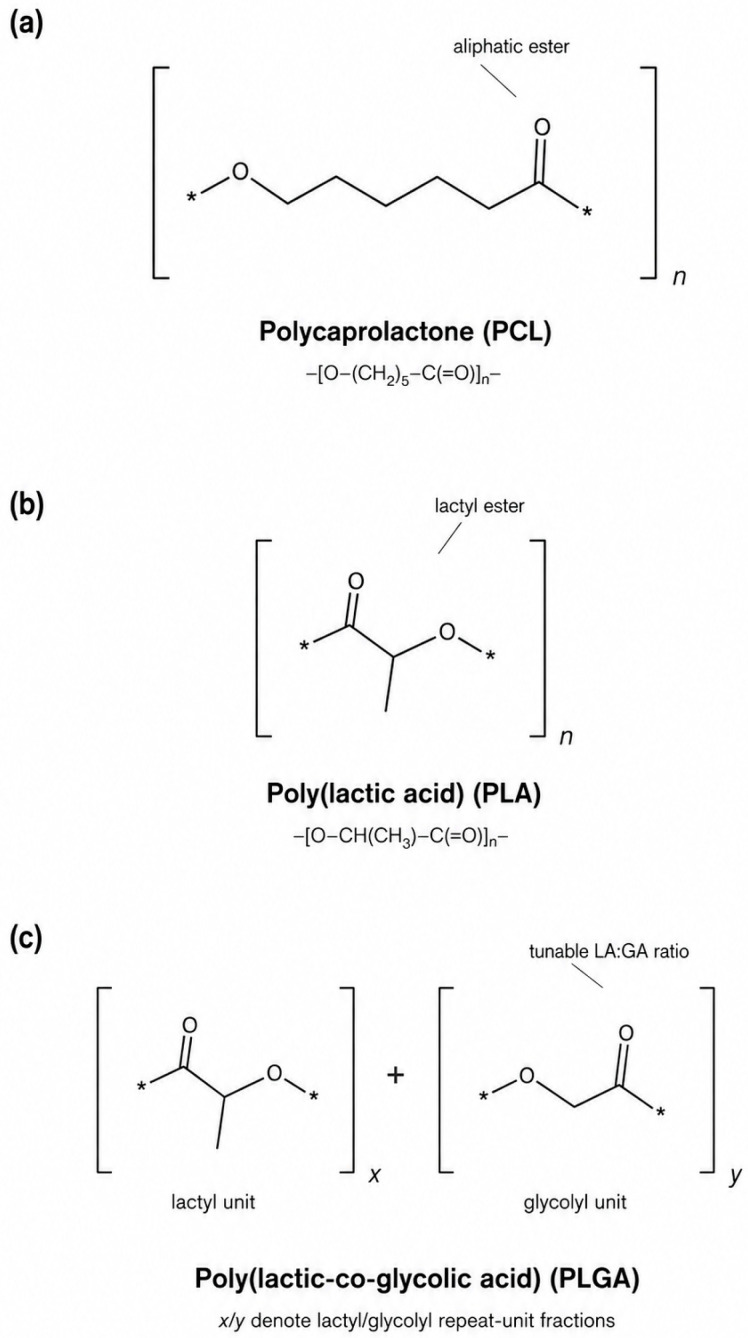
Chemical structures of the synthetic biodegradable polyesters most often blended with biopolymers in 3D-printed bone scaffolds: (**a**) polycaprolactone (PCL); (**b**) polylactic acid (PLA); and (**c**) poly(lactic-co-glycolic acid) (PLGA) with tunable lactyl:glycolyl ratio. The aliphatic ester linkages responsible for hydrolytic degradation are indicated.

The chemical structures of the natural biopolymers discussed in Section 2, and of the synthetic biodegradable polyesters with which they are most often blended, are summarised in Figure 2 and Figure 3, respectively.

### 2.1. Alginate and Gelatin

Alginate, an anionic block copolymer of mannuronic and guluronic acids extracted from brown algae, is the workhorse of extrusion bioprinting because it gels rapidly, mildly, and cytocompatibly through ionic crosslinking with divalent cations such as Ca^2+^, which bridge the guluronate blocks into an “egg-box” network and provide immediate shape retention for cell-laden constructs [16,17]. This fast, reversible gelation, together with low cost and ready availability, accounts for its dominance. Its principal drawback is bio-inertness: alginate lacks mammalian cell-adhesive ligands and osteoinductive cues, and ionically crosslinked networks can lose calcium and destabilise in physiological media over time. The specific media in which this loss occurs are the phosphate- and sodium-rich physiological fluids used for cell culture and implantation—phosphate-buffered saline (PBS), physiological (0.9%) saline, and serum-containing culture media—and the process reflects two coupled ion-exchange mechanisms: monovalent cations, chiefly Na^+^, exchange for the divalent Ca^2+^ that bridges the guluronate blocks and progressively unzip the egg-box junctions, while phosphate (and other chelators such as citrate) sequester the released Ca^2+^ as insoluble calcium phosphate, shifting the equilibrium toward dissolution and accelerating the loss of ionic crosslinks [18]. Alginate is therefore most often blended with gelatin—a denatured, partially hydrolysed collagen that contributes Arg-Gly-Asp (RGD) integrin-binding motifs, enzymatic degradability, and thermoreversible gelation that improves filament definition at reduced temperature [16,19]. The two polymers are complementary: gelatin provides bioactivity and printing viscosity, and alginate provides rapid permanent fixation. Engineered alginate–gelatin inks in which the gelatin fraction is gradually released have been shown to enhance osteogenic differentiation by increasing porosity and progressively exposing adhesion sites, while alginate–gelatin matrices loaded with mesoporous bioactive glass provide sustained ionic release that supports bone repair [19,20]. The alginate–gelatin–hydroxyapatite triad is now among the most studied formulations for printed bone constructs, a deliberate pairing of printability, bioactivity, and mineral reinforcement documented across a growing body of literature [17]. Bibliometric analysis confirms that this combination has become a dominant motif in the field, reflecting both its reliability and the accumulated process knowledge that lowers the barrier to adoption [17]. A recurring refinement is to decouple the printing gel from the permanent network: gelatin is exploited for its thermoreversible viscosity during deposition and then partially released or washed out, leaving a more porous, adhesion-competent alginate scaffold whose increased surface area accelerates osteogenic differentiation [19]. Incorporating an ion-releasing mineral phase such as mesoporous bioactive glass further converts the otherwise inert alginate network into an osteo-stimulatory and sustained-release platform, illustrating how a single blend can be tuned simultaneously for printing behaviour, biological signalling, and drug delivery [20]. Alginate’s versatility also extends to dental and craniofacial contexts, where alginate/gelatin hydrogel scaffolds modulate the proliferation and differentiation of stem-cell populations relevant to mineralised-tissue formation [16].

### 2.2. Gelatin Methacryloyl and Photocrosslinkable Proteins

Functionalising gelatin with methacryloyl groups yields gelatin methacryloyl (GelMA), arguably the most influential bone bioink of the past decade. GelMA retains the cell-adhesive RGD motifs and matrix metalloproteinase-degradable sequences of gelatin while gaining the ability to be covalently photocrosslinked into mechanically stable, hydrolytically tunable networks; stiffness, swelling, and degradation are controlled through the degree of methacryloyl substitution, polymer concentration, photoinitiator, and light dose, giving formulators an unusually wide and independent design space [21,22]. This combination of tunability and intrinsic bioactivity explains its ubiquity. Because GelMA alone is mechanically weak for bone, it is typically reinforced or doped: bioactive-silica GelMA nanocomposites promote stem-cell osteogenic differentiation through silicon release and network stiffening [21], and strontium-doped hydroxyapatite GelMA composites couple a covalent organic network with osteoinductive and anti-resorptive ionic cues [22]. Composite GelMA–polycaprolactone constructs seeded with osteoprogenitor lines have been engineered to enhance both bone formation and vascularisation simultaneously, exploiting the polyester for mechanics and the GelMA for cell instruction [23]. The methacryloyl strategy generalises across the biopolymer family: methacrylated gellan gum has been formulated into bioactive, printable inks for bone tissue-engineered scaffolds [24], and methacrylated gelatin processed into a cryogel and incorporating bioglass supports the regeneration of bone defects through a macroporous, osteoconductive network [21]. Photocrosslinkable proteins thus represent a convergence point between the bioactivity of natural polymers and the spatial precision of light-based fabrication. A practical advantage of the methacryloyl chemistry is that mechanics and degradation can be partially decoupled from polymer identity: raising the degree of substitution or light dose stiffens the network and slows hydrolysis without changing the underlying adhesive protein, allowing a single material to be matched to soft or mineralising niches within one construct [21,22]. This tunability is what enables GelMA to serve simultaneously as the cell-instructive soft phase in hybrid scaffolds and, when reinforced, as a competent osteogenic matrix, and it explains the polymer’s persistence at the centre of bone-bioink research despite its intrinsic mechanical weakness [23].

### 2.3. Collagen

Collagen type I is the dominant organic constituent of native bone matrix and the natural template upon which mineral is deposited, making it the most biologically authentic scaffold material. In practice, however, its weak mechanical properties, rapid enzymatic degradation, batch variability, and poor printability in pure form usually relegate it to the role of a bioactive component within a composite rather than a stand-alone ink [25]. It is therefore commonly combined with synthetic polyesters and minerals that confer structural integrity while preserving its instructive surface chemistry—for instance, in polycaprolactone/collagen/alginate scaffolds incorporating bioactive additives for bone regeneration [25], and in silk fibroin/collagen/hydroxyapatite constructs loaded with osteoanabolic biologics for alveolar bone reconstruction [26]. Gelatin and GelMA, as denatured collagen derivatives that retain the key adhesion motifs while offering far superior processability and thermoreversible or photochemical gelation, are in practice the more printable surrogates for the collagenous phase of bone, which partly explains their predominance over native collagen in printed constructs.

### 2.4. Chitosan

Chitosan, the partially deacetylated derivative of chitin obtained from crustacean shells and fungal cell walls, is a cationic polysaccharide valued for biocompatibility, biodegradability via lysozyme, intrinsic antibacterial activity arising from its positive charge, and structural similarity to the glycosaminoglycans of the natural matrix [8]. These properties, together with its abundance as a by-product of the seafood industry, make it an attractive and sustainable feedstock. Its pH-dependent solubility—soluble only in mildly acidic media—and slow, weak gelation complicate direct printing, so chitosan is most often processed through thermosensitive formulations, chemical derivatisation, or blending with co-polymers. This solubility behaviour is dictated by the pKa of the chitosan primary amine, which is approximately 6.3–6.5 and shifts slightly with the degree of deacetylation and the local charge density along the chain [27]. Below the pKa, the glucosamine amino groups are protonated to ammonium (–NH_3_^+^); the resulting electrostatic repulsion and hydration solubilise the chain in mildly acidic water, whereas as the pH approaches and exceeds the pKa, the amines are progressively deprotonated, interchain hydrogen bonding and hydrophobic association prevail, and the polymer aggregates and precipitates—confining solubility to acidic conditions and limiting direct neutral-pH, cell-laden printing [27]. To enable deposition at the neutral pH that cells require, chitosan is therefore commonly derivatised with hydrophilic substituents that extend solubility into the neutral-to-mildly-alkaline range: carboxymethylation yields carboxymethyl and N,O-carboxymethyl chitosan that dissolve across roughly pH 7–9 without acidic, cytotoxic solvents, while quaternised derivatives (e.g., trimethyl chitosan) and glycol chitosan are likewise water-soluble at physiological pH and have been formulated into printable bioinks [28]. Photocrosslinkable glycol chitosan, for example, enables cell-laden visible-light printing for bone tissue engineering while avoiding the cytotoxicity of ultraviolet exposure [29]. Thermosensitive “green” chitosan–hydroxyapatite scaffolds derived from lyophilised platelet-rich fibrin illustrate a particularly sustainable, growth-factor-rich formulation that combines a natural polymer, a bone-mimetic mineral, and an autologous biologic [30], while chitosan combined with hydroxyapatite and polycaprolactone—including hydroxyapatite recovered from fish scales—demonstrates the valorisation of natural waste streams into osteoconductive, antibacterial composites [31]. Beyond serving as a matrix, chitosan is widely used as a bioactive surface coating on printed scaffolds; microporous chitosan–VEGF coatings, for instance, confer angiogenic capacity onto otherwise inert frameworks [32].

### 2.5. Silk Fibroin

Silk fibroin, extracted principally from *Bombyx mori* cocoons, offers an exceptional and somewhat unusual combination of properties for a natural polymer: high mechanical toughness and tensile strength, tunable proteolytic degradation spanning weeks to years, low immunogenicity, and aqueous processability, which together have established it as a long-standing biomaterial for bone tissue engineering [10,33]. Its mechanical performance derives from controllable β-sheet crystallisation, which acts as a physical crosslink and reinforcing phase and can be modulated through processing to tune stiffness and degradation independently of chemistry; methacrylation further enables photocrosslinkable silk inks compatible with light-based printing [34]. The breadth of printed silk systems reflects this versatility: mesoporous bioactive glass/silk fibroin composites combine osteogenic ion release with a tough matrix [35]; biomimetic silkworm-spinning- and electrohydrodynamic-jet-inspired strategies yield high-strength constructs with ordered, cell-scale fibre architectures that better emulate native matrix organisation [36]; and photocurable nanohydroxyapatite/methacrylated silk fibroin constructs repair calvarial defects in vivo [34]. Increasingly, attention has turned beyond mechanics to the immunomodulatory behaviour of 3D-printed silk proteins, which can steer macrophage polarisation and thereby guide constructive rather than fibrotic bone remodelling—an emerging design lever linking material chemistry to the host response [33]. The processing versatility of silk is itself notable: depending on regeneration conditions and post-treatment, the same protein can be cast as a soft hydrogel; drawn into aligned fibres; or consolidated into a stiff, high-β-sheet solid, so that mechanical properties spanning orders of magnitude are accessible from one sustainable feedstock [10,37]. This breadth allows silk to bridge the soft-to-hard gradient of the osteochondral and cortical environments more readily than most single biopolymers, and underlies its recurrent appearance in both load-relevant composites and interfacial constructs [38,39].

### 2.6. Cellulose and Microbial Biopolymers

Cellulose and its nanoscale forms—cellulose nanofibrils and nanocrystals—are renewable, abundant, and mechanically stiff, and they impart pronounced shear-thinning rheology that markedly improves the printability and post-deposition shape fidelity of otherwise low-viscosity bioinks [11]. Nanocellulose is consequently deployed across many formulations both as a rheology modifier and as a reinforcing phase. Wood-based nanocellulose combined with bioactive glass in gelatin–alginate inks supports the printing and proliferation of bone cells [40], and nanocellulose/chitosan bioinks enhance the osteogenic differentiation of encapsulated cells while improving filament definition [41]. Bacterial cellulose, biosynthesised in high purity as an ultrafine nanofibrous network, contributes mechanical reinforcement and water-holding capacity in polycaprolactone/gelatin/bacterial-cellulose/hydroxyapatite composite scaffolds [42]. Microbial polyesters such as the polyhydroxyalkanoates offer a complementary, fully biodegradable, melt-processable thermoplastic option whose crystallinity and mechanical profile can be tuned through monomer composition to suit load-relevant bone scaffolds, broadening the biopolymer palette beyond hydrogels [43]. More broadly, the systematic dispersion of nanoscale reinforcing phases—nanocellulose, nanosilicates, bioceramic nanoparticles—within a biopolymer matrix to create nanocomposite bioinks has emerged as a defining strategy for reconciling printability with the mechanical and biological function required of bone scaffolds [9,15].

### 2.7. Crosslinking, Modification, and Blending as Unifying Levers

Across all of these feedstocks, three cross-cutting chemical strategies recur and largely determine whether a given biopolymer becomes a usable bone ink. The first is crosslinking, which converts a viscous solution into a shape-stable network and sets the mechanical and degradation envelope; the principal modes—ionic (alginate–Ca^2+^), thermal (gelatin and thermosensitive chitosan), photochemical (GelMA, and methacrylated gellan gum and silk), enzymatic, and physical β-sheet formation in silk—differ in speed, reversibility, and cytocompatibility, and are frequently combined in dual-network designs to obtain both rapid fixation and durable stability [21,24,34]. The second lever is covalent modification, of which methacryloylation is the most influential, since grafting photoreactive handles onto an otherwise thermoresponsive or inert polymer unlocks light-based fabrication and the independent tuning of stiffness and degradation, as realised for gelatin, gellan gum, glycol chitosan, and silk fibroin [22,29]. The third lever is blending and composite formulation, whereby the deficiencies of one component are offset by another—alginate’s bio-inertness by gelatin’s adhesivity, collagen’s weakness by a polyester framework, and a hydrogel’s low stiffness by a dispersed bioceramic or nanomaterial [16,25]. Emerging functional fillers extend this logic beyond mechanics: two-dimensional nanomaterials such as black phosphorus have been incorporated into biopolymer nanoscaffolds to add osteogenic phosphate release and photothermal responsiveness, illustrating how the composite strategy can simultaneously address structure, bioactivity, and stimulus-responsiveness [44]. The practical art of bone-ink design lies in deploying these three levers together so that printability, mechanics, degradation, and bioactivity are tuned without one being sacrificed for another.

## 3. 3D Printing Technologies and Process–Property Relationships

**Figure 4:**
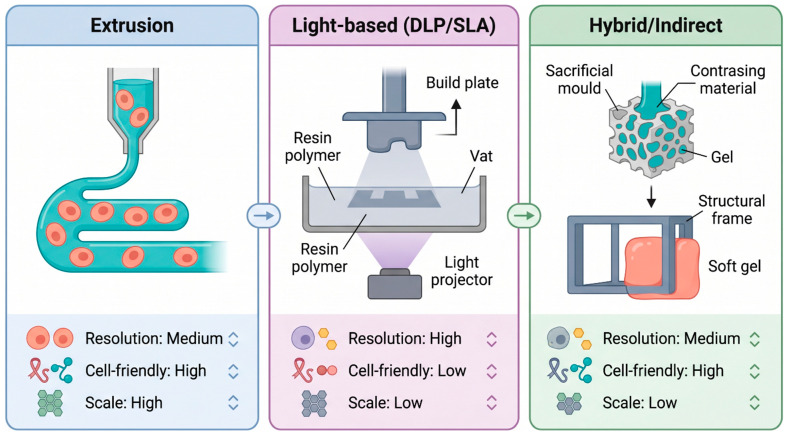
Comparison of extrusion, light-based, and indirect/hybrid printing modalities, with characteristic feedstocks, resolution ranges, and trade-offs.

No single printing modality is optimal for all biopolymers or all bone defects; each imposes distinct constraints on ink rheology, achievable resolution, mechanical outcome, and—where cells are present—viability [5,6]. Matching the fabrication route to the feedstock and clinical target is therefore as consequential as the choice of material itself, and the most capable laboratories increasingly treat material and process as a single co-optimised system rather than as independent choices (Figure 4).

### 3.1. Extrusion-Based Printing and Bioprinting

Extrusion is the most widely used approach for biopolymer bone scaffolds because it accommodates the broadest range of feedstocks—high-viscosity inks, cell-laden hydrogels, and heavily particle-loaded composites—and because it scales readily to clinically relevant dimensions and is comparatively inexpensive [6,7]. A continuous filament is dispensed pneumatically or by mechanical piston or screw and is solidified by thermal gelation, ionic or photochemical crosslinking, or solvent exchange. Printability hinges on shear-thinning behaviour and rapid post-deposition recovery: the ink must thin under the shear of extrusion to pass through the nozzle and then recover sufficient yield stress to support overlying layers without collapse [9]. This rheological requirement is the central reason nanocellulose, gellan gum, and nanoparticulate fillers are routinely added to otherwise poorly printable proteins and polysaccharides, tuning the ink into the narrow window, where it is both extrudable and self-supporting [11,24]. The principal limitations are resolution—typically on the order of one hundred to several hundred micrometres, coarser than the native osteon—and an intrinsic trade-off between cell viability and the shear stress and dispensing pressure required to extrude stiffer, more osteoconductive inks, which can damage encapsulated cells [5]. Advanced extrusion configurations substantially expand the design space. Coaxial and multi-nozzle systems can fabricate osteon-like, hierarchically organised cell-laden constructs and deposit distinct cells or materials in spatial register, enabling core–shell filaments and compartmentalised tissue analogues [45,46]. Embedded or freeform reversible embedding (FRESH) printing dispenses soft, slow-gelling biopolymer inks within a sacrificial support bath that holds the nascent structure until crosslinking is complete, dramatically extending the range of printable low-viscosity formulations, as demonstrated for zoledronic acid-loaded chitosan/alginate/hydroxyapatite thermosensitive hydrogels [47]. The central engineering tension in extrusion is that the same parameters which improve shape fidelity—higher polymer concentration, smaller nozzles, and greater dispensing pressure—also raise the shear stress experienced by encapsulated cells, so that print quality and cell survival pull in opposite directions and must be balanced empirically for each ink [5,9]. Strategies to relieve this tension include adding shear-thinning sacrificial fillers, using conical rather than cylindrical nozzles to reduce peak wall shear, and confining cells to a low-stress core in coaxial filaments while a stiffer shell bears the structural load, the last of which also enables the osteon-mimetic geometries achievable with multi-nozzle systems [11,45].

### 3.2. Light-Based Printing

Vat photopolymerisation and related light-based methods—stereolithography and digital light processing—together with laser- and jet-assisted techniques, cure photoreactive biopolymer resins with high spatial resolution and smooth surface finish, decoupling feature size from nozzle geometry and enabling the reproduction of fine, complex internal architectures [48]. Because resolution is set by the optical system rather than by a physical nozzle, these methods can achieve features finer than extrusion and produce constructs with excellent layer fusion. They are naturally suited to methacryloyl-functionalised biopolymers and other photoreactive resins: photocurable mesoporous-bioactive-glass/tricalcium-phosphate biphasic scaffolds have been printed to mimic vascular channels for bone regeneration [48], visible light-curable glycol chitosan supports cell-laden printing without ultraviolet damage [29], and photocurable nanohydroxyapatite/methacrylated silk fibroin constructs repair cranial defects in vivo [34]. Electrohydrodynamic jet printing, which draws fine jets from a charged meniscus, achieves cell-scale fibre ordering for bone scaffolds that approaches the dimensions of native collagen bundles [36]. The constraints of light-based fabrication include the need for cytocompatible photoinitiators and visible wavelengths to preserve encapsulated cells, the still-limited library of printable biopolymer resins, potential cytotoxicity of unreacted monomer or residual initiator, and the difficulty of incorporating opaque ceramic fillers at high loading without scattering the curing light [48].

### 3.3. Indirect and Hybrid Fabrication

When a target material is intrinsically difficult to print—too stiff, too brittle, or lacking suitable rheology—indirect strategies decouple shaping from the final material composition. Sacrificial moulds defined by 3D printing can be cast against, infiltrated, and then selectively removed, as in light-based printing of leachable salt moulds that template complex porous geometries which would be inaccessible to direct printing of the final material. Hybrid fabrication co-prints a stiff thermoplastic or ceramic framework together with a soft, cell-laden biopolymer phase, combining load-bearing capacity with a biologically instructive interior; magnesium-based nanocomposite frameworks integrated with cell-laden bioinks, for example, yield osteoconductive constructs that neither phase could provide alone [49]. A complementary, modular concept assembles small pre-formed units into larger constructs, granting both manufacturing flexibility and intraoperative adaptability: a 3D-printed, assemblable bespoke scaffold has been used as a versatile microcryogel carrier for site-specific regenerative medicine, allowing cell-laden microgels to be loaded and combined to fit a defect [50], and patient-specific, interlocking “LEGO-inspired” titanium scaffolds illustrate how printed modularity can be exploited to customise geometry at the point of care [51]. These approaches also bridge toward robust, load-bearing implants in which an engineered architecture provides mechanical function, as in 3D-printed PEEK/silicon nitride scaffolds with triply periodic minimal surface structures developed for spinal fusion, where the surface topology tunes both stiffness and osseointegration.

### 3.4. Architecture, Porosity, and Mechanics

**Figure 5:**
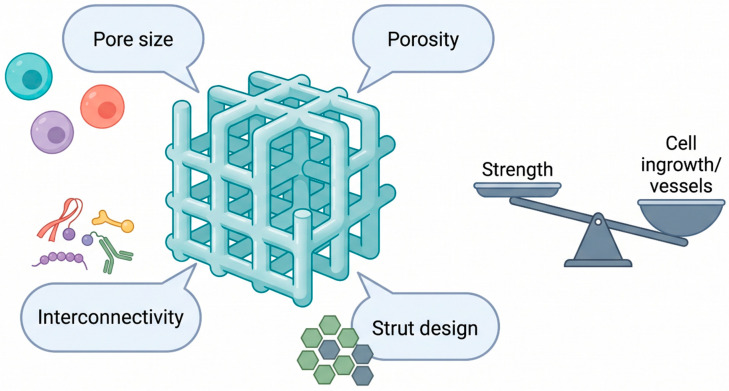
Relationship between printed architecture (pore size, porosity, and strut design) and biological and mechanical outcomes in biopolymer bone scaffolds.

A defining advantage of 3D printing—arguably the advantage that most distinguishes it from conventional fabrication—is deterministic control of internal architecture, which jointly governs biological and mechanical performance. Pore size, total porosity, interconnectivity, tortuosity, and strut geometry regulate cell migration, nutrient and oxygen transport, vascular ingrowth, and the apparent elastic modulus and strength of the construct, and these parameters can now be specified independently of one another within a single design (Figure 5) [4,52]. There is an inherent tension between porosity and strength: higher porosity favours tissue ingrowth and vascularisation but reduces load-bearing capacity, so optimal designs seek a balance appropriate to the site. For bone specifically, the literature converges on an open, fully interconnected pore network with total porosity above roughly 50% and macropore diameters of about 300–400 µm as most favourable for osteoblast migration, vascular ingrowth, and mineralised-matrix deposition, with a practical lower bound near 100 µm, below which osteogenesis is markedly impaired and fibrous or cartilaginous tissue is favoured [53,54]. Systematic comparisons of polycaprolactone/hydroxyapatite scaffolds, for example, demonstrate that bioceramic content and the chosen compounding route—melt blending versus solvent casting—measurably shift both mechanical and biological properties, underscoring that processing history, not only nominal composition, determines outcome [52]. Because biopolymers alone rarely approach the stiffness of cortical or even trabecular bone, architectural design is most powerful when combined with mineral reinforcement, the two acting together to bring the effective modulus toward the host range while preserving an open, osteoconductive, and perfusable pore network [12,13]. Finally, the governing process parameters—nozzle diameter, dispensing pressure, print speed, layer height, crosslinking kinetics, and light dose—must be co-optimised rather than tuned one at a time, since they jointly determine resolution, structural fidelity, and, for cell-laden inks, the viability of the deposited cells [5,6].

### 3.5. Cell Sources and the Biology of Bioinks

When the printed construct is cellularised, the choice and handling of cells become as important as the polymer. Bone bioprinting most often employs mesenchymal stem or stromal cells, which can be sourced from bone marrow or adipose tissue, expanded readily, and differentiated toward the osteogenic lineage by soluble factors, matrix stiffness, and mineral cues [5]. Co-culture strategies that combine osteogenic cells with endothelial or immune cells better recapitulate the cellular ecosystem of healing bone: endothelial cells contribute to vascular-network formation, and macrophages, when appropriately polarised, secrete factors that promote rather than impede osteogenesis, as exploited in dual-channel constructs co-printing macrophages with mesenchymal stem cells [46]. Cell-sourcing innovation can also expand the osteogenic pool from non-classical origins, for example, through transient transcription-factor reprogramming that drives endothelial cells toward an osteogenic phenotype [55] or through the genetic enhancement of stem cells to co-deliver angiogenic signalling, as with VEGF-overexpressing adipose-derived stem cells [56]. Whatever the source, encapsulated cells must survive the mechanical and chemical insults of printing—shear during extrusion, light and radicals during photocuring, and osmotic and ionic shifts during crosslinking—so that maintaining high post-printing viability is a constant design constraint that couples the biology of the bioink to the physics of the process [5,6]. This interdependence is the practical reason that cell type, ink chemistry, and printing modality cannot be selected independently. It also shapes the choice of cell source for translation: autologous stem cells avoid immune rejection but require a second procedure and lengthy expansion, allogeneic sources ease scale-up at the cost of immunological matching, and genetically enhanced or reprogrammed cells add potency but raise regulatory complexity, so the cell-sourcing decision propagates directly into manufacturing and approval pathways [46,55,56]. For acellular composite scaffolds, these constraints largely disappear, which is one reason such constructs are likely to reach the clinic earlier than their cell-laden counterparts even when the latter show superior regeneration in animal models [14].

### 3.6. Biocompatibility and Surface-Mediated Cell–Scaffold Interactions

Because the clinical value of any printed construct ultimately depends on how host cells and tissues respond to it, biocompatibility is best treated as an explicit design output rather than an assumed property of natural polymers. For 3D-printed biopolymer scaffolds, it is assessed across a hierarchy of endpoints: in vitro cytocompatibility—viability, adhesion, proliferation, and osteogenic differentiation of osteoblasts and mesenchymal stem cells—and in vivo host response, comprising the absence of chronic inflammation or fibrous encapsulation, controlled and predictable degradation, and progressive osseointegration. Systematic evaluation of printed scaffolds in femoral and tibial defect models confirms that well-designed biopolymer and biopolymer–ceramic constructs support robust bone regeneration with a favourable host response, and that this performance is coupled as much to architecture—interconnected porosity above ~50% with 300–400 µm macropores—as to chemistry [54].

The earliest events at the scaffold–tissue interface are physicochemical and largely electrostatic: serum proteins adsorb within seconds, and the amount, identity, and conformation of this adsorbed layer—which in turn dictates integrin-mediated cell attachment—are governed by the surface charge, wettability, and roughness of the material [57]. Surface charge, conveniently captured by the zeta potential, is therefore a meaningful predictor of cell behaviour, and the biopolymers reviewed here span a broad range: from cationic, positively charged chitosan, whose protonated amines promote electrostatic adhesion of negatively charged cell membranes and confer intrinsic antibacterial activity, to the anionic, negatively charged alginate, gelatin near physiological pH, silk fibroin, and cellulose derivatives. Moderate positive surface potential together with nanoscale-to-micron roughness generally enhances osteoblast adhesion, spreading, and osteogenic differentiation, whereas strongly electronegative or very smooth surfaces tend to support weaker attachment, so that blending, polyelectrolyte complexation, mineral incorporation, and surface functionalisation are routinely used to tune interfacial charge and topography toward an osteoconductive response [57]. Reporting zeta potential and surface roughness alongside the usual mechanical and rheological metrics would allow these structure–response relationships to be compared across studies and would strengthen the mechanistic interpretation of both in vitro and in vivo outcomes [57].

## 4. Composite and Functionalisation Strategies

**Figure 6:**
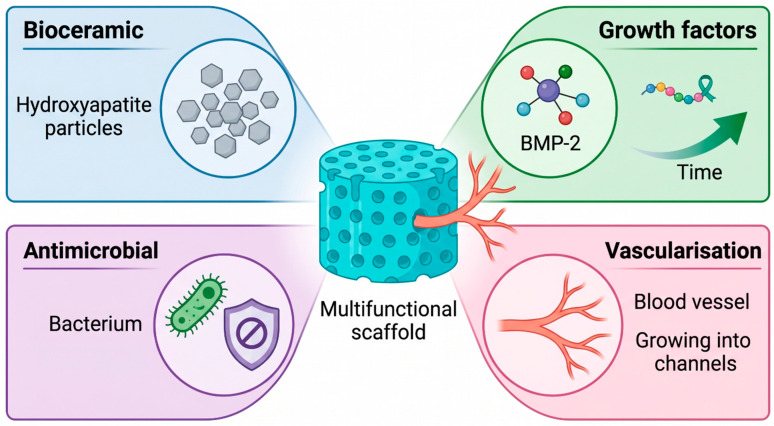
Composite and functionalisation strategies: bioceramic reinforcement, growth-factor and gene delivery, vascularisation, and antimicrobial/multifunctional design.

The transition from a merely printable biopolymer to a genuinely osteogenic scaffold is achieved largely through composition and functionalisation. Two complementary levers dominate: incorporation of inorganic phases to provide stiffness and osteoconductivity, and the controlled presentation of biological or pharmacological signals to direct cell behaviour, recruit host cells, and protect the construct from infection and disease recurrence [9,12]. The most advanced scaffolds combine both, and they increasingly do so with spatial and temporal precision (Figure 6).

### 4.1. Biopolymer–Bioceramic Composites

Native bone is itself a polymer–ceramic nanocomposite of collagen and carbonated apatite, and the most successful printed scaffolds mirror this organisation by dispersing a mineral phase within a biopolymer matrix to recreate both its mechanics and its chemistry [2,12]. Calcium phosphates—hydroxyapatite and β-tricalcium phosphate foremost—are the dominant fillers because of their compositional similarity to bone mineral, their osteoconductivity, and the release of osteogenic calcium and phosphate ions during gradual dissolution; their ratio can be tuned to balance long-term stability against resorption, and biphasic calcium phosphate formulations are widely used to exploit this balance [58]. Hydroxyapatite reinforcement appears in many guises: biomimetic hydroxyapatite mineralised onto nanoclay/polycaprolactone scaffolds for cranial repair [59] and strontium-substituted nanohydroxyapatite composites in which the substituent ion adds anti-resorptive and pro-osteogenic signalling beyond simple osteoconduction [22,60]. Bioactive glasses and related inorganic phases dissolve to release silicon, calcium, and other ions that stimulate both osteogenesis and angiogenesis, and they have been printed both as stand-alone bioceramic scaffolds and as fillers within biopolymer matrices [20,58,61]. Beyond the calcium phosphates, silicon nitride has emerged as a versatile and somewhat unconventional bioceramic for bone applications: silicon nitride-reinforced cryogels combine osteogenic activity with intrinsic antibiofilm behaviour, addressing mechanics and infection simultaneously [62]; silicon nitride composites with biopolymers and elastomers broaden the accessible property range, including damping behaviour relevant to physiological load transfer [62]; and comparative in vitro studies of the cellular and osteogenic response to alternative biomaterials inform rational bioceramic selection for spinal and other load-bearing implants. Across all of these systems, the inorganic–biopolymer composite is the central design motif for reconciling the bioactivity and processability of natural polymers with the stiffness and osteoconductivity demanded by bone [12]. Beyond mechanics, the dissolution products of the mineral phase are themselves bioactive: calcium and phosphate ions support matrix mineralisation, while substituent and dopant ions such as strontium, magnesium, and silicon modulate the balance of bone formation and resorption and stimulate angiogenesis, so the ceramic functions simultaneously as reinforcement and as a sustained ionic drug [22,60,61]. A subtler design goal is to match the resorption of the mineral to that of the polymer and to the rate of new-bone formation; mismatches leave either a persistent ceramic residue that impedes remodelling or a prematurely weakened construct, which is why tunable biphasic calcium phosphates and controllable silicon nitride systems are attractive, as their dissolution can be engineered rather than merely accepted [62,63].

### 4.2. Growth-Factor and Gene Delivery

Osteoinduction is most directly achieved by delivering morphogens—foremost bone morphogenetic protein-2 (BMP-2)—from the printed scaffold to recruit and differentiate progenitor cells. The clinical experience with BMP-2, however, has shown that bolus or supraphysiological delivery risks ectopic bone formation, inflammation, and soft-tissue swelling, so spatiotemporal control of release is essential to both efficacy and safety [64]. Printed scaffolds have accordingly been engineered for the dual delivery of BMP-2 and human mesenchymal stem cells to regenerate critical-size defects, co-locating the inductive signal and the responsive cell population [64]. Biopolymer cryogel systems extend this principle by releasing multiple growth factors in a defined temporal sequence designed to recapitulate the natural healing cascade, in which angiogenic and osteogenic signals act in succession rather than simultaneously [65]. Macroporous hybrid scaffolds with sustained drug delivery similarly couple durable structural support to prolonged biological signalling, decoupling the timescales of mechanics and release [13]. More sophisticated programmable platforms co-deliver neurotrophic and osteogenic cues—for example, microsphere–hydrogel scaffolds that release nerve growth factor and BMP-2-mimetic peptides to restore innervation alongside bone formation, reflecting growing recognition that functional bone requires a nerve supply, as well as a blood supply [66]. Gene- and cell-level strategies complement protein delivery and can provide more durable signalling: transient overexpression of OCT-4 facilitates BMP4-induced osteogenic transdifferentiation of endothelial cells, expanding the pool of osteogenic cells from a vascular source [55], and VEGF-overexpressing adipose-derived stem cells combined with whitlockite-reinforced cryogels couple a vasculogenic genetic cue to a magnesium-bearing osteoconductive mineral to enhance bone regeneration [56]. Finally, cell-instructive composite bioprinting can deliberately engage host immunity, as in dual-channel constructs that combine bone marrow-derived macrophages with mesenchymal stem cells to coordinate early immune regulation with osteogenic induction, treating the immune response as a designable input rather than an obstacle [46].

### 4.3. Vascularisation Strategies

Insufficient vascularisation is the foremost biological obstacle to regenerating defects larger than a few millimetres, beyond which passive diffusion can no longer sustain cells in the construct interior, leading to a necrotic core [67]. Printing is uniquely positioned to address this problem because it can pattern vasculature both structurally and biologically. Spatially patterned bioprinting of distinct osteogenic and vasculogenic regions creates pre-organised templates that guide the co-development of bone and its blood supply [67], while biphasic or channelled architectures incorporate hollow conduits that mimic vascular channels and accelerate host-vessel ingrowth [48]. Composite bioinks have been formulated explicitly for vascularised bone: gelatin/gellan-gum scaffolds with double-crosslinked networks balance printability, mechanics, and a permissive environment for vessel formation [68], and GelMA/polycaprolactone constructs have been shown to enhance concurrent osteogenesis and angiogenesis [23]. Angiogenic functionalisation provides a biochemical complement to architecture, including microporous chitosan–VEGF coatings that present a pro-angiogenic factor at the surface [32] and deferoxamine-releasing strontium-hydroxyapatite scaffolds that stabilise hypoxia-inducible-factor signalling to drive angiogenesis pharmacologically [69]. Biopolymer carriers can also be engineered for the vasculature directly, as in heparin-functionalised injectable cryogels with rapid shape-recovery designed to sequester and present heparin-binding angiogenic factors for neovascularisation [70]. The current frontier is the bioprinting of prevascularised bone organoids, which bring a nascent, self-organised vascular network to the defect so that anastomosis with the host circulation—rather than slow ingrowth—drives rapid in situ reconstruction [71]. The biological imperative behind these efforts is the roughly 200-micrometre diffusion limit of oxygen, beyond which cells in a construct interior become hypoxic and die unless a perfusable network is established quickly; this single constraint explains why vascularisation, not osteogenesis, is usually the rate-limiting step in scaling from millimetre to centimetre defects [67]. The most convincing strategies therefore couple a structural template for large vessels—printed channels or biphasic conduits—with biochemical cues that recruit and stabilise the microvasculature, such as surface-presented VEGF or hypoxia-mimetic deferoxamine, and increasingly with co-printed endothelial or organoid units that supply the network directly rather than relying on host invasion [32,48,69]. The convergence of these structural, biochemical, and cellular approaches, rather than any one alone, is what distinguishes the more recent and more successful vascularised-bone constructs [23,68].

### 4.4. Antimicrobial and Multifunctional Scaffolds

Infection is a leading cause of bone-graft and implant failure, and treating an infected defect conventionally requires staged surgery; printed biopolymer scaffolds increasingly integrate antimicrobial function alongside osteogenesis to address both problems in one construct. The rate and duration of release from these printed constructs are set by a combination of mechanisms that scaffold design can deliberately bias—Fickian diffusion of the agent through the hydrated matrix and interconnected pores, swelling-controlled release as the network hydrates and relaxes, and erosion- or degradation-controlled release as the biopolymer is hydrolytically or enzymatically resorbed—so that surface-adsorbed drug typically gives an early burst, whereas matrix-encapsulated or microsphere-carried drug yields more sustained, near-zero-order profiles; strut spacing, porosity, and the partitioning of drug between dense and porous regions or into secondary carriers thus become direct levers on the release profile [72]. Drug-eluting strategies dominate and include the local release of antibiotics—vancomycin-loaded sustained-release microspheres in dual-nozzle nanohydroxyapatite scaffolds [73], linezolid-carrying nanohydroxyapatite/PLGA constructs for infected defects [74], and zoledronic acid-loaded chitosan/alginate/hydroxyapatite hydrogels that combine an anti-resorptive agent with a mineralised matrix [47]—as well as bifunctional hydrogel-integrated scaffolds that simultaneously clear infection and support repair through a single integrated design [75]. Material-level infection resistance can avoid added drugs altogether: the intrinsic antibacterial character of cationic chitosan and the antibiofilm activity of silicon nitride confer protection through the scaffold chemistry itself [8,62]. For oncological defects, where tumour recurrence and bone loss must be addressed together, multifunctional scaffolds couple ablative therapy with regeneration, as in near-infrared-programmable shape-memory scaffolds that enable combined bone-tumour management and repair through photothermal control [76], and multifunctional polyetheretherketone bone scaffolds engineered to treat osteosarcoma and osteomyelitis in parallel [77]. Collectively, these designs reflect a broader and accelerating shift from passive, purely structural scaffolds toward responsive, multifunctional, stimuli-active implants tailored to the full clinical context of a defect [3].

## 5. Applications and Translational Performance

**Figure 7:**
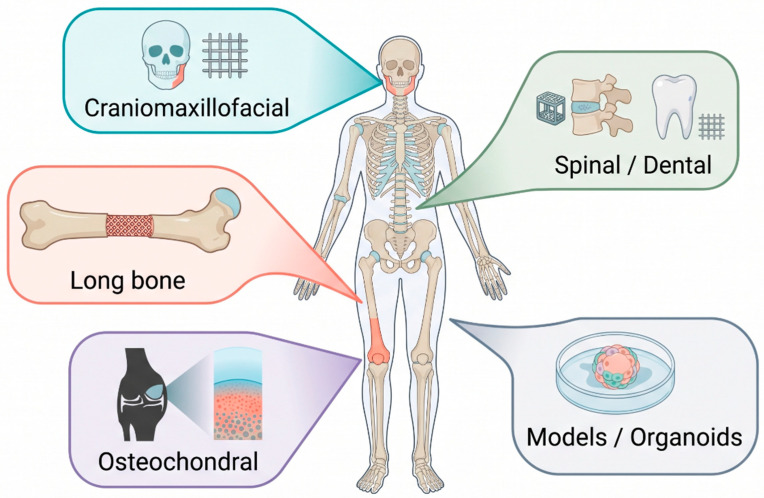
Applications of 3D-printed biopolymer scaffolds across craniomaxillofacial, load-bearing, osteochondral, spinal/dental, and disease-model contexts, with representative preclinical outcomes.

The strategies above are ultimately judged by their performance in physiologically relevant defects. This section surveys representative applications by anatomical and functional context (Figure 7) and weighs the preclinical evidence that will inform clinical translation, noting both achievements and the recurring gaps that limit comparability across studies; representative preclinical studies are summarised in Table 2.

### 5.1. Craniomaxillofacial and Non-Load-Bearing Defects

Calvarial, cranial, and alveolar defects are common proving grounds for printed biopolymer scaffolds because they are geometrically complex, frequently non-load-bearing, and exceptionally well served by patient-specific fabrication, where exact anatomical fit governs both function and appearance. Printed biopolymer composites have successfully repaired calvarial and cranial defects in rodent models using several distinct chemistries: biomimetic hydroxyapatite mineralised on nanoclay/polycaprolactone [59], photocurable nanohydroxyapatite/methacrylated silk fibroin [34], and immunomodulatory dual-channel constructs that combine macrophages with mesenchymal stem cells to steer the early healing environment [46]. In alveolar reconstruction, silk fibroin/collagen/hydroxyapatite scaffolds loaded with an osteoanabolic biologic improved bone formation in a clinically pertinent oral context [26]. The convergence of patient-specific geometry with osteoinductive composition makes craniomaxillofacial repair the most clinically mature application area for printed biopolymer scaffolds, and the relatively forgiving mechanical demands of these sites allow the bioactivity of natural polymers to be exploited with less compromise than in load-bearing bone [3].

### 5.2. Load-Bearing and Long-Bone Defects

Load-bearing sites impose the dual requirement of osteoconduction and durable mechanical support, exposing the central weakness of soft biopolymers and motivating the composite and hybrid designs discussed above. Critical-size long-bone regeneration has been demonstrated with printed scaffolds delivering BMP-2 together with mesenchymal stem cells in a rabbit tibia model, where the combination of inductive signal and responsive cells drove robust bridging [64]. Encouragingly, the field is advancing toward larger, more clinically representative models: 3D-printed cryogel-impregnated functionalised scaffolds have been shown to augment the healing of a critical tibial fracture in a goat, an important step beyond the small-animal models that dominate the literature and a better surrogate for human loading and defect scale [78]. Hybrid constructs that co-print stiff frameworks with cell-laden biopolymers, including magnesium-based nanocomposites, directly target the simultaneous provision of load-bearing capacity and biological activity that single-phase materials cannot achieve [49]. A systematic appraisal of polylactic-acid/bioceramic composite scaffolds across preclinical in vivo studies indicates a consistent osteoconductive benefit, while at the same time underscoring the heterogeneity of defect models, follow-up periods, and outcome metrics that complicates quantitative comparison and meta-analysis across the field [14]. Personalised, signal-enriched constructs point toward the next stage of individualised therapy, exemplified by bioprinted scaffolds that combine injectable platelet-rich fibrin with laponite nanoclay to drive efficient regeneration through defined signalling pathways tuned to the patient [79]. The persistent obstacle in this domain is the gap between the small-animal models that supply most of the evidence and the mechanical and biological demands of human load-bearing bone; rodent calvarial and femoral defects rarely reproduce the cyclic loading, defect volume, and slower healing of a human long bone, so the comparatively rare large-animal studies carry disproportionate translational weight [14,78]. This is also where pure biopolymers are least sufficient and where hybrid and modular designs—stiff printed frameworks infilled with cell-laden gels, or assemblable units combined to fill a defect—are most clearly motivated, since they supply the immediate mechanical competence that soft natural polymers cannot while preserving an instructive biological interior [49,50,51].

### 5.3. Osteochondral and Interfacial Repair

Restoration of the osteochondral unit poses a distinct challenge because it requires spatially graded constructs that transition continuously from articular cartilage at the surface to mineralised subchondral bone beneath—a task to which multi-material and multi-zone printing is uniquely suited. Biphasic and gradient scaffolds developed for this purpose include silk-infilled biphasic constructs whose mechanical and physical properties are tuned across the two zones for osteochondral tissue engineering [39], alongside the wider range of scaffold materials and fabrication techniques that have been reviewed specifically for the osteochondral interface [80]. Crucially, success at the interface depends on guiding tissue maturation rather than merely supporting it: bioceramic-mediated control of chondrocyte hypertrophy has been used to promote calcified-cartilage formation for osteochondral defect repair in a rabbit model, directing the cellular programme toward the calcified transition zone that couples cartilage to bone. The unifying requirement across these efforts is the printing, within a single construct, of continuous gradients in composition, stiffness, and porosity that avoid the mechanical and biological discontinuities responsible for the failure of earlier bilayered approaches [80].

### 5.4. Spinal and Dental Applications

Two further clinical domains illustrate the breadth of printed biopolymer and composite scaffolds. In spinal surgery, interbody fusion requires implants that bear substantial cyclic load while encouraging bony bridging, and printed architectures with engineered surface topology have been explored to balance these demands, as in PEEK/silicon nitride scaffolds with triply periodic minimal surface structures designed for spinal fusion [81]. Rational material selection for such load-bearing implants is informed by comparative in vitro studies of the cellular and osteogenic response to alternative biomaterials, which help identify combinations that favour osseointegration over fibrous encapsulation. In dentistry and oral surgery, printed biopolymer constructs serve both regenerative and cell-delivery roles: alginate/gelatin hydrogel scaffolds influence the proliferation and differentiation of dental pulp stem cells [16], and chitosan–hydroxyapatite formulations enriched with platelet-rich fibrin provide sustainable, autologously sourced grafts for alveolar and periodontal repair [30]. These domains reinforce a recurring lesson: as mechanical demand rises, the design centre of gravity shifts from soft biopolymer hydrogels toward reinforced composites and hybrid architectures, while the biopolymer continues to supply the biological interface [2].

### 5.5. Disease Models and Organoids

Beyond reconstruction, printed biopolymer constructs increasingly serve as three-dimensional in vitro models of bone biology and disease that recapitulate native architecture and cell–matrix interactions far more faithfully than two-dimensional culture, improving the physiological relevance of mechanistic and pharmacological studies [82]. Such biomimetic bone models support the study of tumour–bone interactions, metastasis, and treatment response in a tissue-mimetic context, and engineered models of tumour and bone have been developed specifically to understand tumour-induced bone disease and to improve therapy [82]. The bioprinting of prevascularised bone organoids extends this trajectory from model to therapy, generating self-organising, vascularised bone units that can serve both as research platforms and as implantable grafts for rapid in situ reconstruction [71]. Decellularised extracellular matrix, which retains tissue-specific composition and a complex repertoire of bioactive cues difficult to reconstitute synthetically, provides a complementary bioink for both the modelling and the regeneration of bone and cartilage [83]. Efforts to engineer matrices that emulate the liquid-crystalline ordering and viscoelastic mechanics of the natural bone extracellular matrix further refine the biological fidelity of printed constructs, recognising that cells respond not only to composition and stiffness but also to matrix dynamics and molecular order [84]. These modelling applications carry a practical significance beyond basic science: physiologically faithful printed bone constructs offer a route to higher-throughput, more predictive drug and toxicity testing that could reduce reliance on animal experiments and shorten development timelines, provided the models can be standardised and produced reproducibly [82,85]. The same biopolymer and bioceramic toolkit that builds implantable scaffolds thus also builds research platforms, and advances in one domain—better bioinks, finer architecture, and more reliable vascularisation—propagate to the other, reinforcing the case for treating material, process, and biological design as a unified programme rather than a set of application-specific recipes [9,15].

## 6. Challenges and Future Perspectives

Despite rapid and broad-based progress, several interlocking challenges continue to separate current biopolymer bone scaffolds from routine clinical use, and they are best understood as coupled rather than independent problems. Foremost is vascularisation. Although patterned printing, channelled architectures, angiogenic functionalisation, and prevascularised organoids have each advanced the field, the reliable generation of a hierarchical, perfusable, and rapidly host-integrating vascular network within clinically sized constructs remains unsolved, and it is the principal reason that scaffold performance so often degrades as defect volume increases from the small-animal to the human scale [67,71]. Closely linked is the matching of mechanical properties and degradation kinetics to the host site. Biopolymers seldom meet the stiffness and fatigue resistance of load-bearing bone, and although bioceramic reinforcement and architectural design narrow this gap, the independent control of degradation remains difficult: a scaffold that resorbs faster than bone forms loses mechanical integrity prematurely, whereas one that degrades too slowly impedes remodelling and can provoke chronic inflammation [12,52].

A second cluster of obstacles concerns manufacturing and standardisation. The batch-to-batch variability of naturally sourced biopolymers—arising from differences in species, harvest, and extraction—complicates reproducibility and regulatory approval, motivating tighter sourcing, purification, and characterisation standards. This agenda is well aligned with the principles of sustainable, green biomaterials production and is exemplified by the deliberate valorisation of natural waste streams, such as the recovery of hydroxyapatite from fish scales, which converts a disposal problem into a high-value osteoconductive feedstock [8,31]. Scaling cell-laden bioprinting to clinical dimensions while preserving cell viability, sterility, and consistent quality across a construct large enough to fill a human defect remains a formidable manufacturing problem, compounded by the limited shelf life of living inks and the difficulty of terminal sterilisation of a cellularised product. At the level of evidence, the heterogeneity of preclinical defect models, animal species, follow-up durations, and outcome metrics hampers cross-study comparison, meta-analysis, and the construction of the robust efficacy dossiers that regulators require [5,14]. Part of the remedy is methodological rather than material: consistent reporting of ink rheology, crosslinking conditions, printed architecture, mechanical properties under physiologically relevant loading, and degradation in vivo would allow the many promising single studies to be compared and combined, and would expose which design choices generalise across systems [12,52]. The sustainability agenda intersects here as well, since reproducible, well-characterised feedstocks—including those recovered from natural waste streams—are a prerequisite for both regulatory confidence and the green-chemistry credentials that motivate biopolymer use in the first place [8,31].

Regulatory and translational considerations deserve explicit attention because they increasingly determine which laboratory advances reach patients. A printed, cell-laden, drug-releasing biopolymer scaffold may simultaneously meet the definitions of a medical device, a biologic, and a combination product, placing it within a complex and still-evolving regulatory framework that demands clear specification of starting materials, manufacturing-process controls, and release criteria. Acellular composite scaffolds—bioceramic-reinforced polyesters and printed biopolymer–mineral constructs—face a comparatively lower translational barrier than cell-laden bioprinted tissues and are likely to reach broad clinical use sooner, which argues for parallel development tracks rather than a single roadmap. Cost, supply-chain robustness, point-of-care versus centralised manufacturing, and the integration of printing into the surgical workflow are practical determinants of adoption that are easy to overlook in materials-focused research yet decisive in practice. Encouragingly, modular and assemblable designs that allow standardised units to be combined or customised intraoperatively offer one route to reconcile personalisation with manufacturable, inspectable components [50,51].

**Figure 8:**
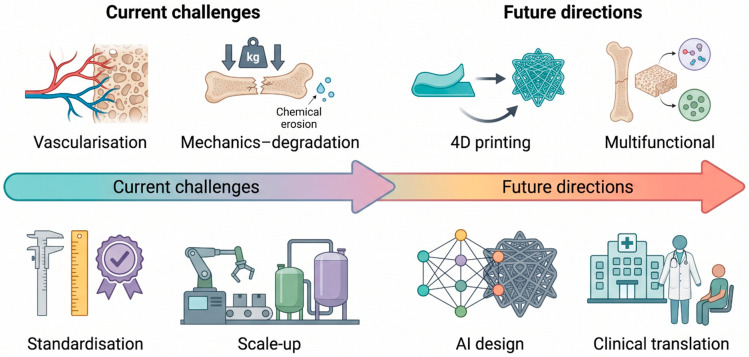
Roadmap of challenges and future directions: Vascularisation; mechanical–degradation matching; manufacturing and regulatory standardisation; and emerging 4D, multifunctional, and data-driven design.

Future directions are likely to be defined by greater intelligence, responsiveness, and integration in design (Figure 8). Four-dimensional printing, in which a printed construct changes shape or function over time in response to physiological stimuli, is exemplified by near-infrared-programmable shape-memory bone scaffolds that can be deployed minimally invasively and then actuated in situ [76]. A closely related direction is in situ (intraoperative) bioprinting, in which bioink is deposited directly into the defect during surgery rather than printed in vitro and implanted; handheld and robotic in situ printers conform the construct to the wound in real time, reduce contamination risk, and exploit the native microenvironment for maturation [86,87]. The personalisation of such constructs is increasingly driven by clinical imaging: high-resolution computed-tomography and magnetic-resonance datasets are segmented and converted, through computer-aided design, into patient-specific geometries with anatomically exact external form and internal porosity gradients, closing the loop between diagnostic imaging and point-of-care fabrication [86]. Multifunctional and stimuli-responsive scaffolds that couple regeneration with infection control, tumour therapy, or reinnervation point toward implants tailored to the complete clinical context of a defect rather than to osteogenesis alone [66,75]. Computational and data-driven approaches to ink formulation and architecture optimisation—including machine learning-guided exploration of the large composition–process design space—together with modular and hybrid fabrication that deliberately combines the strengths of multiple materials and processes, offer credible routes to constructs that are simultaneously printable, mechanically competent, and biologically instructive [50,51]. Realising these advances will depend on the continued co-design of material chemistry, fabrication process, and biological function that has characterised the most successful work to date, and on closer alignment between the laboratory, the clinic, and the regulator [2,3].

## 7. Conclusions

Three-dimensional printing of biopolymer-based scaffolds has matured into a versatile and sustainable platform for bone tissue engineering. Natural polymers supply biodegradability, biocompatibility, and extracellular matrix-mimicking cues; additive manufacturing supplies patient-specific geometry and deterministic internal architecture; and composite and functionalisation strategies supply the mechanical competence and biological signalling that bone regeneration demands. The clearest lesson of the literature is that the most effective scaffolds are those in which feedstock chemistry, printing process, and biological design are developed together rather than optimised in isolation, since a gain in one dimension is frequently purchased at the expense of another. Persistent challenges—vascularisation, mechanical and degradation matching, manufacturing reproducibility, and regulatory standardisation—define a clear and shared research agenda. Addressing them through intelligent, multifunctional, and integrated design, supported by sustainable sourcing and standardised evaluation, will be decisive in translating biopolymer 3D printing from compelling preclinical demonstrations into dependable, widely available clinical bone repair. The trajectory of the field suggests that the decisive advances will come not from any single breakthrough material or printer but from the disciplined integration of modest, well-characterised gains across the whole design chain—an ink that prints reliably, a mineral that resorbs in step with new bone, a vascular template that perfuses promptly, and a manufacturing route that a regulator can inspect—assembled into constructs that are at once sustainable, reproducible, and biologically convincing [2,3,12].

## Figures and Tables

**Table 1:** Principal biopolymer feedstocks for 3D-printed bone scaffolds.

Biopolymer	Source	Crosslinking	Mechanical Character	Degradation	Typical Role
Alginate	Brown algae	Ionic (Ca^2+^)	Weak, brittle gel	Slow, ion-exchange–dependent	Fast-gelling extrusion base; blended with gelatin
Gelatin/GelMA	Hydrolysed collagen	Thermal; photo (methacryloyl)	Tunable, soft–moderate	Enzymatic, tunable	Cell-adhesive bioink; photopatternable
Collagen	Animal tissue	Thermal/enzymatic	Weak	Rapid, enzymatic	Bioactive component in composites
Chitosan	Crustacean/fungal chitin	pH/thermal; photo (derivatives)	Moderate, brittle	Lysozyme-mediated	Antibacterial matrix and coating
Silk fibroin	*Bombyx mori* cocoons	Physical (β-sheet); photo (methacryloyl)	Tough, high strength	Proteolytic, tunable weeks–years	Load-relevant matrix; immunomodulatory
Cellulose/nanocellulose	Plant, bacterial	Physical; additive reinforcement	Stiff nanofibres	Slow (non-mammalian enzymes)	Rheology modifier and reinforcement
Polyhydroxyalkanoates	Microbial fermentation	Melt/thermoplastic	Tunable, thermoplastic	Hydrolytic/enzymatic	Biodegradable load-bearing framework

**Table 2:** Representative preclinical studies of 3D-printed biopolymer-based bone scaffolds.

Biopolymer System	Printing Method	Defect Model	Key Outcome	Ref.
PLA–Biogel + BMP-2/hMSC	Extrusion	Rabbit tibia, critical size	Dual delivery drove robust bridging	[64]
Cryogel-impregnated functionalised scaffold	Extrusion + cryogel	Goat tibia, critical fracture	Augmented healing at large-animal scale	[78]
Nano-HA/methacrylated silk fibroin	Light-based (photocuring)	Rat calvaria	Enhanced osteogenesis and defect repair	[34]
Biomimetic HA on nanoclay/PCL	Extrusion	Rat cranium	Improved mineralisation and bone formation	[59]
Silk fibroin/collagen/HA + EPO	Extrusion	Alveolar bone	Increased bone formation in oral context	[26]
Gelatin/gellan gum, double-crosslinked	Extrusion bioprinting	Vascularised bone construct	Concurrent osteogenesis and angiogenesis	[68]
Prevascularised bone organoid	Bioprinting	Cranial reconstruction	Rapid in situ vascularised bone formation	[71]
PCL/SrHA@DFO	Extrusion	Bone defect	Coupled regeneration and vascularisation	[69]
Macrophage + BMSC, dual-channel	Extrusion bioprinting	Rat calvaria	Immune regulation plus osteogenic induction	[46]

## Data Availability

No new data were created or analyzed in this study. Data sharing is not applicable to this article.
